# Study protocol of the DUtch PARkinson Cohort (DUPARC): a prospective, observational study of de novo Parkinson’s disease patients for the identification and validation of biomarkers for Parkinson’s disease subtypes, progression and pathophysiology

**DOI:** 10.1186/s12883-020-01811-3

**Published:** 2020-06-13

**Authors:** Jeffrey M. Boertien, Sygrid van der Zee, Asterios Chrysou, Marleen J. J. Gerritsen, Nomdo M. Jansonius, Jacoba M. Spikman, Teus van Laar, the PPNN Study Group, N. A. Verwey, B. Van Harten, A. T. Portman, M. J. H. Langedijk, P. G. Oomes, B. J. A. M. Jansen, T. Van Wieren, S. J. A. Van den Bogaard, W. Van Steenbergen, R. Duyff, J. P. Van Amerongen, P. S. S. Fransen, S. K. L. Polman, R. T. Zwartbol, M. E. Van Kesteren, J. P. Braakhekke, J. Trip, L. Koops, C. J. De Langen, G. De Jong, J. E. S. Hartono, H. Ybema, A. L. Bartels, F. E. Reesink, A. G. Postma, G. J. H. Vonk, J. M. T. H. Oen, M. J. Brinkman, T. Mondria, R. S. Holscher, A. A. E. Van der Meulen, A. W. F. Rutgers, W. A. Boekestein, L. K. Teune, P. J. L. Orsel, J. E. Hoogendijk, T. Van Laar

**Affiliations:** 1 Department of Neurology, University Medical Center Groningen, University of Groningen, P.O. Box 30.001, 9700RB Groningen, The Netherlands; 2 Parkinson Expertise Center Groningen, Groningen, the Netherlands; 3 Department of Neuropsychology, University Medical Center Groningen, University of Groningen, Groningen, the Netherlands; 4 Department of Ophthalmology, University Medical Center Groningen, University of Groningen, Groningen, the Netherlands

**Keywords:** Parkinson disease, Neurodegenerative diseases, Observational study, Longitudinal studies, Biomarkers, Neuropsychology, Cognition, Gastrointestinal microbiome, Microbiota, Ophthalmology

## Abstract

**Background:**

Parkinson’s Disease (PD) is a heterogeneous, progressive neurodegenerative disorder which is characterized by a variety of motor and non-motor symptoms. To date, no disease modifying treatment for PD exists. Here, the study protocol of the Dutch Parkinson Cohort (DUPARC) is described. DUPARC is a longitudinal cohort study aimed at deeply phenotyping de novo PD patients who are treatment-naïve at baseline, to discover and validate biomarkers for PD progression, subtypes and pathophysiology.

**Methods/design:**

DUPARC is a prospective cohort study in which 150 de novo PD subjects will be recruited through a collaborative network of PD treating neurologists in the northern part of the Netherlands (Parkinson Platform Northern Netherlands, PPNN). Participants will receive follow-up assessments after 1 year and 3 years, with the intention of an extended follow-up with 3 year intervals. Subjects are extensively characterized to primarily assess objectives within three major domains of PD: cognition, gastrointestinal function and vision. This includes brain magnetic resonance imaging (MRI); brain cholinergic PET-imaging with fluoroethoxybenzovesamicol (FEOBV-PET); brain dopaminergic PET-imaging with fluorodopa (FDOPA-PET); detailed neuropsychological assessments, covering all cognitive domains; gut microbiome composition; intestinal wall permeability; optical coherence tomography (OCT); genotyping; motor and non-motor symptoms; overall clinical status and lifestyle factors, including a dietary assessment; storage of blood and feces for additional analyses of inflammation and metabolic parameters. Since the start of the inclusion, at the end of 2017, over 100 PD subjects with a confirmed dopaminergic deficit on FDOPA-PET have been included.

**Discussion:**

DUPARC is the first study to combine data within, but not limited to, the non-motor domains of cognition, gastrointestinal function and vision in PD subjects over time. As a de novo PD cohort, with treatment naïve subjects at baseline, DUPARC provides a unique opportunity for biomarker discovery and validation without the possible confounding influences of dopaminergic medication.

**Trial registration:**

NCT04180865; registered retrospectively, November 28th 2019.

## Background

Parkinson’s disease (PD) is the second most prevalent neurodegenerative disorder, affecting up to 1 in 100 adults over the age of 60 [1]. Despite its large societal impact, the cause of PD remains elusive and only symptomatic treatments exists that mainly targets the motor symptomatology. In addition, the clinical diagnosis of PD poses a clear diagnostic challenge and is rejected in 20% of the cases [2, 3]. Though PD is clinically defined by motor symptoms resulting from dopaminergic neurodegeneration in the substantia nigra, non-motor symptoms are present in the majority of cases and often precede the motor symptoms by years [4]. The constellation of both motor and non-motor symptoms greatly differs between PD subjects, making PD a heterogeneous disorder in which different clinical subtypes might represent different etiologies [5, 6].

To advance our understanding of PD pathophysiology and move towards disease modifying treatments, it is essential to investigate PD as broad as possible, taking into account its large clinical heterogeneity. For this purpose, extensive clinical characterization and biomarker assessments are required for adequate classification of PD subtypes and associated prognostic and pathophysiological markers. Ideally, studies designed to discover and validate biomarkers in PD should only include treatment-naïve PD subjects at baseline, to avoid the possible confounding effect of dopaminergic medication. However, such study cohorts are sparsely available.

This paper describes the study protocol of the DUtch PARkinson Cohort (DUPARC), a single-center, prospective, longitudinal, observational, cohort study at the University Medical Center Groningen (UMCG) of de novo PD subjects, who are treatment-naïve at baseline. PD participants are extensively characterized with the aim to discover and validate biomarkers for PD subtypes, progression and pathophysiology. Though the endpoints assessed in the DUPARC study allow for subject characterization across a wide variety of domains, DUPARC focuses on three domains in particular, representing the interests and expertise of our research group: (1) cognition, (2) gastrointestinal function and (3) vision.

### Cognition

Cognitive dysfunction is a common non-motor symptom in PD that greatly influences the quality of life of patients. Mild cognitive impairment (PD-MCI) is already present in 25–30% of newly diagnosed patients and is considered an important risk factor for the development of PD dementia [7, 8]. The cognitive profile in PD is highly heterogeneous, with multiple cognitive domains affected.

The underlying pathology of cognitive impairment in PD is complex and includes the degeneration of multiple neurotransmitter systems, of which the cholinergic system is thought to be of particular importance [9–11]. In vivo cholinergic imaging studies revealed cholinergic deficits in PD patients compared to control subjects, with the cholinergic loss being even more pronounced in PD patients with dementia, suggesting a direct relationship between cholinergic denervation and cognitive decline in PD [12–14]. However, these findings are based on indirect Positron Emission Tomography (PET) imaging with a tracer binding to acetylcholinesterase. Although acetylcholinesterase is considered to be a reliable target for cholinergic imaging, it is located both on pre- and postsynaptic membranes, and also binds postsynaptically on non-cholinergic neurons [15, 16]. Recently, the selective PET tracer [^18^F]Fluoroethoxybenzovesamicol (FEOBV) was introduced and validated [17, 18]. This tracer binds presynaptically to the vesicular acetylcholine transporter and is a more sensitive and regional cholinergic marker compared to the cholinesterase binding tracers [17, 18].

Combining selective dopaminergic and cholinergic imaging with detailed neuropsychological assessment in a longitudinal cohort of de novo, treatment-naïve PD patients, provides the opportunity to explore the exact role of the cholinergic system in specific cognitive domains, to identify sensitive biomarkers of cognitive decline, and explore future therapeutic targets. We hypothesize that (1) in newly diagnosed, treatment-naïve PD patients, a significant proportion of patients will show cognitive impairment related to regional cortical and subcortical cholinergic denervation; (2) baseline regional cholinergic denervation will be a predictor of cognitive decline in PD over time.

### Gastrointestinal function

Gastrointestinal dysfunction, in particular constipation, is one of the earliest manifestations of PD and can occur up to 20 years before diagnosis [19]. In concordance with the symptomatology, alpha synuclein deposition in Lewy bodies and neurites, the pathological hallmark of PD, are also found in the Enteric Nervous System (ENS) of PD cases [20, 21]. The alpha synucleinopathy is believed to spread in a prion-like manner via the vagal nerve to the brain [22]. Moreover, intestinal inflammation and an increased intestinal wall permeability are found in PD [23]. Possible determinants of the enteric pathology in PD include gut microbiota, which were found to modulate the synucleinopathy, neuroinflammation and motor symptomatology in a rodent PD model [24].

Recently, 16 independent studies have shown that the gut microbiota composition of PD patients is significantly different from healthy age-matched controls (HC) [25]. Interestingly, the gut microbiome changes in PD do not match those in idiopathic constipation, despite the high prevalence of constipation in PD [26]. So far, published studies on gut microbiota composition in PD almost exclusively included dopamine-suppleted PD patients, which is a significant confounder. Moreover, subtype analyses within PD have been limited, in which PD subjects with autonomic dysfunction, indicative of a possible gut-first subtype, might be of particular interest [27]. Therefore, to advance the development of gut microbiota composition as a biomarker or possible therapeutic target in PD, it is a requirement to establish the gut microbiota composition of extensively characterized treatment-naïve PD patients.

We hypothesize that (1) gut microbiome composition of treatment-naïve de novo PD subjects is different from HC; (2) dopaminergic medication drives differential abundance of gut microbial taxa independent of PD progression; (3) PD subjects with autonomic dysfunction, indicative of a gut-first subtype, will show distinct gut microbial signatures compared to other PD subjects; (4) the gut microbiome changes in (constipated) PD subjects will be different from the changes in idiopathic constipation subjects; (5) the intestinal wall permeability will be increased already in de novo PD subjects with concomitant signs of intestinal inflammation.

### Vision

PD presents with a broad range of visual system dysfunctions, including visual hallucinations and defects in color vision, contrast sensitivity and visual fields [28–32]. Along with visual symptoms, thinning of the retinal nerve fiber layer can be assessed by optical coherence tomography (OCT) and is observed in PD, including thinning of the inner-plexiform and the ganglion cell layer [33]. The pattern of retinal thinning and the peripheral visual defects in PD bear resemblance with glaucoma, a disorder with a pathophysiological and epidemiological link with PD [34, 35]. Though retinal thinning has been suggested as possible biomarker for PD diagnosis, severity and progression [32, 36], our understanding of the visual system in PD, and the possible link with pathologies such as glaucoma, is still limited.

Two studies have been published investigating the retina in de novo treatment-naive patients with OCT [37, 38], and reported statistically significant thinning of the retinal nerve fiber layer and macular ganglion cell-inner plexiform layer. In treated PD subjects, it has been shown that dopaminergic treatment and other interventions commonly applied in PD, such as deep brain stimulation, can alleviate some of the visual symptoms present in PD, but are also able to induce or worsen visual symptoms [30]. However, there is a lack of longitudinal studies incorporating the effect of dopaminergic treatment in PD. In order to understand the effects of PD on visual function and retinal structure, including the seemingly contradictory effects of PD medication, there is a need for an in-depth ophthalmological assessment in a longitudinal PD cohort, with data collection before and after treatment initiation.

We hypothesize that (1) de novo PD patients will show a specific pattern of retinal thinning, in particular affecting the inner plexiform layer; (2) the retinal thinning pattern of PD will be different from glaucoma, with retinal changes focusing on the inner plexiform layer in PD, contrary to the ganglion cell layer in glaucoma; (3) dopaminergic medication will increase the thickness of retinal cell layers, especially the inner plexiform layer; (4) the severity of retinal thinning will be a predictor of PD disease progression.

## Methods/design

### Study design

The DUPARC study is a single center, prospective, observational study of 150 de novo PD patients who are treatment naïve at baseline with follow-up after 1 year and 3 years, with the intention of an extended follow-up with three-year intervals. DUPARC is designed to assess 16 specific objectives with a focus on the domains of cognition, gastrointestinal function and vision, with the overarching aim to discover and validate biomarkers for PD subtypes, progression and pathophysiology (Table 1).

**Table 1:** Objectives of the DUPARC cohort study

**Overall aim**	
To discover and validate biomarkers for Parkinson’s disease (PD) subtypes, progression and pathophysiology.	
**General**	
1.	To combine clinical assessment of both motor and non-motor symptoms with outcome measures across multiple domains, including neuropsychology, gastroenterology and ophthalmology.
**Cognition**	
2.	To establish the relationship between cognitive impairment and cholinergic innervation in treatment-naïve PD patients.
3.	To determine the relationship between cognitive impairment and dopaminergic innervation in treatment-naïve PD patients.
4.	To investigate the progression of the cognitive profile of de novo PD patients and determine the incidence of PD associated mild cognitive impairment over time.
5.	To identify potential biomarkers of longitudinal cognitive decline within the brain neurotransmitter system.
6.	To identify potential biomarkers of longitudinal cognitive decline within the brain functional connectivity and white matter tracts using functional MRI and diffusion tensor imaging.
**Gastrointestinal function**	
7.	To establish the gut microbiome composition of treatment-naïve PD subjects compared to age- and sex-matched control subjects.
8.	To determine the possible influence of dopaminergic medication on gut microbiome composition in PD after 1 year of dopaminergic medication use.
9.	To determine the specificity of gut microbiome changes for PD diagnosis by additionally correcting for possible confounders other than dopaminergic medication, eg. dietary habits, presence and severity of constipation, non-dopaminergic medication, disease history.
10.	To identify potential biomarkers of PD within the gut microbiome, ranging in complexity from the identification of key microbes, suitable for rapid quantification, to complex microbial fingerprints. Potential biomarkers of PD should be further validated for their specificity compared to other neurological and neurodegenerative disorders, as well as their robustness in other PD microbiome studies.
11.	To correlate changes in gut microbiome composition in PD to specific PD subtypes in terms of clinical presentation, rate of progression, genetic risk profile and/or imaging parameters.
12.	To investigate the gut permeability of treatment-naïve PD subjects through the assessment of fecal and serum markers, as well as a urinary sugar excretion test.
**Vision**	
13.	To determine retinal cell layer thickness in treatment-naïve PD subjects compared to age- and sex-matched control subjects.
14.	To determine retinal cell layer thickness in treatment-naïve PD subjects compared to age- and sex-matched glaucoma subjects.
15.	To determine clinical correlates of structural retinal changes and functional tests, including visual, motor, non-motor and neuropsychological scores.
16.	To determine the effect of dopaminergic medication on retinal layer thickness and visual function.

### Study population

#### In- and exclusion criteria

Inclusion criteria consist of PD diagnosis by a movement disorders specialist according to Movement Disorder Society Clinical Diagnostic Criteria for PD, with a confirmed dopaminergic deficit by means of an 3,4-dihydroxy-6-18F-fluoro-l-phenylalanine (^18^F-FDOPA) PET scan.

PD subjects who are unable to provide written informed consent, who are unable to comply with study procedures, who have exclusion criteria for Magnetic Resonance Imaging (MRI), who have gastrointestinal exclusion criteria influencing gut microbiome composition, or whose PD diagnosis is rectified during follow-up, are excluded from the study cohort. A detailed overview of the applicable in- and exclusion criteria is provided in Supplementary Table 1.

#### Recruitment strategy

Participants are recruited via Parkinson Platform Northern Netherlands (PPNN), a collaborative network of PD treating neurologists in 13 medical centers in the northern part of the Netherlands. PPNN serves a total of approximately 5000 PD patients, with an incidence of 400 newly diagnosed PD patient within this network. Treatment-naïve de novo PD subjects who are willing to participate are referred to the University Medical Center Groningen (UMCG) to assess the in- and exclusion criteria. After inclusion and completion of the baseline assessments, patients return to their treating neurologist to continue care as usual, including the start with dopaminergic treatment.

#### Control cohorts

Though the complete DUPARC protocol solely concerns de novo PD subjects, there is ample opportunity to interpret the assessed endpoints relative to appropriate control cohorts. Primarily, age- and sex-matched control subjects are recruited separately to ensure identical assessment techniques for the neuropsychological, gastroenterological and ophthalmological assessments, representing the three main domains of the protocol. In addition, appropriate control subjects for the ophthalmological assessments, including HC and over 1000 glaucoma patients, are available under a different IRB-protocol in the UMCG. These all received the ophthalmological assessments, including retinal imaging, according to the UMCG standard operating procedures, using the same OCT machine, during overlapping periods, to ensure maximal compatibility with the DUPARC data. Complementary, the gut microbiome and genetic assessments will be compared to control subjects from LifeLines, a three generation, population-based cohort study concerning 167.000 participants from the same geographical area in the northern part of the Netherlands [39]. Of these participants, biomaterials such as fecal samples and blood samples have been collected in two large subcohorts (*n* = 9.000 and *n* = 1.100) [40, 41]. In addition, 38.000 LifeLines participants, of a total of 53.000 participants of whom genotype data is available, have received the same genome-wide genetic assessment using the Illumina GSA-MD chip. Genotype information will eventually become available for all 167.000 LifeLines participants. To ensure compatibility, the LifeLines standard operating procedures for the gut microbiome and genetic analyses are also used in DUPARC. Biomaterial from selected LifeLines participants can be reanalyzed to assess possible batch effects. Moreover, stool frequency and consistency has been assessed using the same stool diary in DUPARC and LifeLines, allowing for the identification of LifeLines subjects with idiopathic constipation. Lastly, a large proportion of endpoints, including the neuropsychological assessments, will be assessed using norm-rated tools that depict the performance of the patient as a percentile corrected for relevant demographics, such as age and level of education.

### Study procedures

**Fig. 1:**
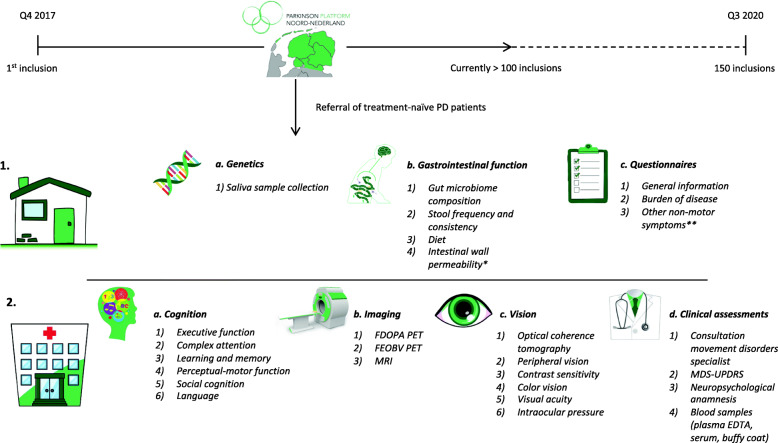
Baseline recruitment and assessments from Q4 2017 to Q3 2020 of treatment-naïve de novo PD subjects through the collaborative network of PD treating neurologists Parkinson Platform Northern Netherlands (PPNN). 1. Study procedures start at home with 1a. the collection of a saliva sample for genetic screening; 1b. Assessments of gastrointestinal function and stool sample collection; 1c. questionnaire assessments. 2. Participants visit the University Medical Center Groningen (UMCG) on 2 days for 2a. a complete cognitive assessment; 2b. imaging; 2c. ophthalmological assessments; 2d. clinical assessments. * In a subset of participants, intestinal wall permeability will also be assessed using blood samples and a urinary excretion test. ** Hyposmia is also assessed using the Sniffin’ sticks. Source clipart: clipart-library.com

At baseline, study procedures start at home with the collection of biomaterials for genetic screening and the completion of gastroenterological assessments and various other questionnaires. After the home assessments, participants visit the UMCG during 2 days for PET and MR-imaging, as well as a complete neuropsychological, ophthalmological and clinical assessment. An overview of the baseline assessments is depicted in Fig. 1.

Participants will receive follow-up assessments after 1 and 3 years, with the intention of an extended follow-up with three-year intervals. After 1 year, participants are visited at home for a repeated gastroenterological, clinical and questionnaire assessment. During the three-year follow-ups the complete baseline assessments are repeated with the exception of the genetic and gastrointestinal assessments. All assessed endpoints and the time of assessments are presented in Table 2.

**Table 2:** Overview assessments DUPARC

Assessments	Endpoint	Baseline	Follow-up 1 year	Follow-up 3 years
**Cognition**				
Montreal cognitive assessment	Cognitive screening	X	X	X
Rey auditory verbal learning test	Learning and memory	X		X
Location learning test	Learning and memory	X		X
Wechsler Adult Intelligence Scale IV: Digit Span	Learning and memory	X		X
Wisconsin card sorting test	Executive functioning	X		X
Letterfluency	Executive functioning	X		X
Hayling sentence completion test	Executive functioning	X		X
Stroop color word test	Complex attention	X		X
Trail making test	Complex attention	X		X
Vienna Test System Reaction time test	Complex attention	X		X
Boston naming test	Language	X		X
Semantic Fluency	Language	X		X
Test of everyday attention: Map search	Perceptual-motor function	X		X
Judgment of line orientation	Perceptual-motor function	X		X
Facial expression of emotion: Stimuli and tests	Social cognition	X		X
Dutch adult reading test	Premorbid intelligence	X		X
**Vision**				
Farnsworth D15, Lanthony D15	Color vision	X		X
Optical Coherence Tomography	Structural retinal imaging	X		X
HFA2 SITA Fast	Peripheral vision	X		X
Pelli Robson Contrast Sensitivity	Contrast sensitivity	X		X
Non-contact tonometry	Intraocular pressure	X		X
Visual acuity	Visual acuity	X		X
**Gastrointestinal function**				
16S rRNA gene and metagenomic sequencing	Fecal microbiome composition	X	X	
Stool diary (7 days)	Stool frequency and consistency	X	X	
Dietary diary (3 days)	Nutrient intake	X	X	
Fecal calprotectin	Intestinal wall permeability - inflammation	X		
Fecal alpha1-antitrypsin	Intestinal wall permeability - protein leakage	X		
Serum zonulin^a^	Intestinal wall permeability - mucosal barrier integrity	X		
Urinary sugar excretion test^a^	Intestinal wall permeability	X		
Serum LPS^a^	Intestinal wall permeability - microbial translocation	X		
**Other non-motor symptom assessment**				
Sniffin’ Sticks	Hyposmia	X		X
Non Motor Symptom Questionnaire	Non-motor symptoms screening	X	X	X
REM Sleep Behavioral Disorder Questionnaire	REM sleep behavioral disorder	X	X	X
Hospital Anxiety and Depression Scale	Anxiety and depression	X	X	X
Dutch Multifactor Fatigue Scale	Fatigue	X	X	X
Apathy Evaluation Scale	Apathy	X	X	X
Questionnaire for Impulsive-Compulsive Disorder in Parkinson’s Disease Rating Scale	Impulsive-compulsive disorder	X	X	X
**Motor assessment**				
MDS-UPDRS III	Motor functioning	X	X	X
Hoehn & Yahr	Disease severity	X	X	X
MDS-UPDRS IV	Motor complications dopaminergic medication		X	X
**Burden of disease**				
MDS-UPDRS II	Motor aspects of experiences of daily living	X	X	X
Utrechtse coping lijst	Coping	X	X	X
Parkinson’s Disease Quality of Life Questionnaire 39	Quality of life	X	X	X
Dysexecutive Questionnaire	Dysexecutive syndrome	X	X	X
Zarit Caregiver Burden inventory	Caregiver burden	X	X	X
**General information**				
Disease History		X	X	X
Demographics		X		X
**Imaging**				
FDOPA PET	Brain dopaminergic imaging	X		X
FEOBV PET	Brain cholinergic imaging	X		X
MRI brain – Resting State		X		X
MRI brain – Diffusion Tensor Imaging		X		X
MRI brain – Arterial Spin Labeling		X		X
MRI brain – T1		X		X
MRI brain – T2		X		X
MRI brain - Susceptibility Weighted Imaging		X		X
**Genetics**				
GSA-MD	Genome-wide genotyping	X		
**Blood samples**^a^				
Plasma EDTA (8 × 2 ml aliquots)		X		X
Serum (4 × 2 ml aliquots)		X		X
Buffy coat (2x)		X		X

*EDTA* Ethylenediaminetetraacetic acid, *FDOPA PET* 18Fluor dopamine positron emission tomography, *FEOBV PET* 18Fluoroethoxybenzovesamicol positron emission tomography, *GSA-MD* Illumina Infinium Global Screening Assay (MD variant), *HFA2 SITA Fast* Humphrey Field Analyzer 2 SITA 24–2 – Fast visual field perimetry, *LPS* Lipopolysaccharide; *MDS-UPDRS* Movement Disorders Society Unified Parkinson’s Disease Rating Scale, *MoCA* Montreal Cognitive Assessment, *MRI* Magnetic Resonance Imaging

^a^Blood withdrawal and urinary sugar excretion tests are performed in a subset of participants

### Data management

Data are stored in an electronic Case Report Form (eCRF) using Castor, a Good Clinical Practice (GCP) certified electronic Data Capture (EDC). Data unfit for storage in the eCRF (eg. imaging and metagenomic data) are stored on local secured servers at the UMCG. The metadata are stored in the eCRF. Study monitoring will be performed by in-house study monitors of the UMCG. Depending on the type of data and associated privacy regulations, data from the DUPARC project will be made publicly available or will become available via the corresponding author, upon reasonable request.

### Sample size

Since most objectives of the DUPARC cohort study concern a first assessment in treatment-naïve de novo PD subjects, a formal power calculation is hampered, as no adequate estimation of the expected effect sizes can be provided. However, the effect sizes previously reported in already treated PD subjects with longer disease duration are mostly expected to be larger than the effect sizes in our cohort. Therefore, a power calculation based on previous reports will provide us with a minimum number of participants to include, to assess the most important endpoints within the domains of cognition, gastrointestinal function and vision.

The primary objective related to the cognitive domain is to establish the relationship between cognitive impairment and pre-synaptic cholinergic degeneration in de novo PD patients. This will be analyzed using cholinergic PET-imaging with FEOBV in combination with neuropsychological assessments covering all cognitive domains, including complex attention, learning and memory, executive function, perceptual-motor function, language, and social cognition. Because of the novelty of FEOBV, previous research using FEOBV-PET in PD is limited. Significant results were found in a small group comparing PD and control subjects [18]. Cross-sectional comparisons between PD subgroups have included between 15 and 79 PD patients, with significant results [42]. The size of the DUPARC cognition data is therefore expected to be sufficient for group and sub-group analyses, as well as detailed correlational research on the specific cognitive domains. Because of the great heterogeneity at baseline in both cognitive performance and cortical cholinergic innervation in PD, such a large cohort is needed for correlational analyses [43, 44].

The main objective within the gastrointestinal function domain is the comparison of the gut microbiome composition with HC. Though a power calculation for metagenomic analyses is not possible, we think our sample size will be sufficient to do these analyses, because previous studies already reported significant differences with far smaller sample sizes varying between 10 and 72 included PD subjects. Only two treatment-naïve subgroups of 12 and 39 PD subjects have been investigated in previous studies, reporting fewer taxonomic differences in the treatment-naïve group, compared to already treated subjects. The DUPARC gut microbiome data therefore surpasses most PD gut microbiome studies in sample size, and is by far the largest treatment-naïve dataset, allowing for more robust microbiome composition signatures and correction for confounders.

Regarding the domain of vision, the most recent meta-analysis on OCT imaging in PD has reported overall mean effect sizes of 0.45 for several retinal cell layers, comparing HC to PD subjects [33]. For a similar effect size, with an alpha of 0.05, and a two-tailed comparison of means, a sample size of 79 subjects for each group would be sufficient to achieve a power of 0.80.

The sample size of 150 participants therefore clearly surpasses the expected numbers needed to assess the primary objectives within the key domains of DUPARC. Nevertheless, a sample size of 150 participants is still necessary, as the anticipated effect sizes of various biomarkers in treatment-naïve de novo PD subjects is expected to be lower, compared to treated PD subjects with longer disease duration. In addition, to find biomarkers indicative of PD subtypes, the PD sample not only has to provide both case and control samples for a subtype comparison, but also the intergroup differences are expected to be lower if PD subgroups are compared. Our sample size of 150 participants will most likely allow for binary and trinary subtype comparisons, given the aforementioned samples sizes required to distinguish PD from HC. Lastly, the sample size also needs to be larger than calculated to account for participants who will be lost to follow-up. For this purpose, 180 participants will be included at baseline to account for 30 drop-outs and non-PD cases, in order to ensure a sample size of 150 participants.

### Statistical analysis

Relevant statistical analyses will be performed dependent on the applicable objective as formulated in Table 1. Since the various endpoints within and between domains could be densely correlated, especially based on the hypothesis that PD characteristics aggregate within distinctive PD subtypes, the correlations of variables across domains will be assessed [45]. Provided the extensiveness and complexity of the DUPARC dataset, advanced statistical techniques, including artificial intelligence, will be used for cross-domain analyses.

### Ethics approval and consent to participate

DUPARC is conducted in accordance with the Declaration of Helsinki and national and international standards of GCP. Potential participants receive detailed written and oral information on the study procedures and all participants provide written informed consent. Ethical approval of the study protocols was obtained from the Medical Ethics Review Board of the University Medical Center Groningen (METc UMCG). The DUPARC study protocol is registered at the Dutch Central Committee on Research Involving Human Subjects (CCMO) with trial registration number NL60540.042.17, whereas the microbiome study is registered separately with trial registration number NL61123.042.17. In addition, the study has been registered retrospectively on November 28th 2019 at clinicaltrials.gov, with identifier NCT04180865.

### Study timetable

The first visit of the first patient was performed in Q4 2017. Baseline inclusion is planned until Q3 2020, and is on schedule. The milestone of 100 included PD subjects with a confirmed dopaminergic deficit on FDOPA-PET was reached in Q3 2019. The first publications on the baseline data are expected in 2020, followed by the first follow-up data in 2023.

## Discussion

DUPARC will be the first study to assess a variety of biomarkers and associated endpoints, combining multiple important disease domains, including cognition, gastrointestinal function, vision, neuroimaging and motor performance, in a cohort of de novo PD subjects who are treatment naïve at baseline. Therefore, DUPARC provides a unique opportunity for biomarker discovery. Complementary, DUPARC can be used to validate previous findings from existing cohort studies in a treatment-naïve PD cohort.

There is a large need for early markers of PD, its subtypes and progression. First, disease-modifying interventions should ideally be implemented as soon as possible to slow down the course of the disease, highlighting the need for a more accurate diagnosis of PD in the early phases of the disease. Second, given the clinical and possible etiological heterogeneity of PD, adequate subject stratification is imperative to advance our understanding of the disease mechanisms and informs trial design for putative disease-modifying trials. Third, the development of disease-modifying therapies is hampered by the lack of a solid measurement of disease progression. DUPARC fulfills these needs, given the inclusion of early stage de novo PD subjects, and the long-term follow-up with extensive assessment of endpoints in a variety of non-motor and motor domains of PD. The included patients in DUPARC are therefore very suitable for disease-modifying trials.

Biomarker discovery in PD should ideally be performed in treatment-naïve PD patients at baseline, since this eliminates the possible confounding effects of dopaminergic medication that cannot adequately be corrected for in a case-control setting. Causative inferences can be summarized by three possible scenarios, in which the biomarker is either causative for PD, caused by PD or is a confounder. Therefore, biomarkers discovered in the DUPARC treatment-naïve de novo PD cohort provide promising leads for replication and validation in relevant cohorts, and functional inquiry into the pathophysiological significance of the biomarker and its potential as a therapeutic target. Such validation studies might include PD cohorts enriched for a specific PD associated genotype (eg. *GBA1* mutations) or functional research on patient derived biomaterial by reprogramming of induced pluripotent stem cells (eg. organ-on-a-chip, human intestinal organoids).

Additionally, DUPARC has an optimal design with its single center set-up and the extensive assessment of endpoints. Though participants are recruited from 13 medical centers, all study assessments are performed in the UMCG or at home by UMCG investigators, according to the same standard operating procedures. This allows for the combined analysis of endpoints across various domains, without possible confounding influences of interinstitutional and interrater variability. Next to addressing the objectives of DUPARC as formulated in Table 1, this cohort provides multiple opportunities to combine endpoints across various domains, such as gastrointestinal dysfunction and cholinergic denervation, which will further advance our understanding of the disease mechanisms of PD and its subtypes.

Lastly, DUPARC can be used to validate the findings of other cross-sectional or longitudinal PD cohorts. Since most cohort studies concern already treated patients or lower numbers of treatment- naïve subjects, validation of their results is required to disentangle the putative confounding influence of dopaminergic medication from any PD related effects. A considerable overlap can be observed with other cohort studies such as the Personalized Parkinson Project and the Parkinson’s Progression Markers Initiative, giving ample opportunity for cross-linking and validation of findings in our single-center study cohort of treatment-naïve PD subjects. In addition, the relation of more extensively studied domains with relatively unique endpoints in DUPARC, such as retinal layer scanning and cholinergic denervation, can be assessed.

In conclusion, DUPARC provides a unique opportunity for biomarker discovery and validation for PD subtypes, progression and pathophysiology in a treatment-naïve de novo PD cohort across multiple important disease domains.

## Supplementary information

**Additional file 1: Supplementary Table 1.** Exclusion criteria DUPARC.

## Data Availability

Depending on the type of data and associated privacy regulations, data from the DUPARC project will be made publicly available or will become available via the corresponding author, upon reasonable request.

## Abbreviations

CCMO: Central Committee on Research Involving Human Subjects
eCRF: electronic Case Report Form
DUPARC: DUtch PARkinson Cohort
EDC: Electronic Data Capture
ENS: Enteric Nervous System
FDOPA: 3,4-dihydroxy-6-18F-fluoro-l-phenylalanine
FEOBV: [^18^F]Fluoroethoxybenzovesamicol
GCP: Good Clinical Practice
HC: Healthy control(s)
MCI: Mild cognitive impairment
MRI: Magnetic Resonance Imaging
OCT: Optical coherence tomography
PD: Parkinson’s disease
PET: Positron Emission Tomography
PPNN: Parkinson Platform Northern Netherlands
UMCG: University Medical Center Groningen
