# Supplementary material for: Study protocol of the DUtch PARkinson Cohort (DUPARC): a prospective, observational study of de novo Parkinson’s disease patients for the identification and validation of biomarkers for Parkinson’s disease subtypes, progression and pathophysiology
Source: BMC Neurol. 2020 Jun 13;20:245. doi: 10.1186/s12883-020-01811-3 (PMC7293131; doi:10.1186/s12883-020-01811-3)
Supplement: Supplementary file 1 — Additional file 1: Supplementary Table 1. Exclusion criteria DUPARC. [file 12883_2020_1811_MOESM1_ESM.docx]

| **Supplementary table 1** Exclusion criteria DUPARC | |
| --- | --- |
| **General** |  |
| - Inability to provide written informed consent and/or comply with study procedures - Dopaminergic medication use - Refusal to be informed about an unforeseen clinical finding | |
| **Gastrointestinal function**   - Active or persistent primary disease of the gastrointestinal tract - History of peritonitis, severe endometriosis, abdominal, intestinal or urogenital fistula - Hepatobiliar or pancreatic disease (except asymptomatic cholecystolithiasis) - History of abdominal or anorectal surgery, except minor surgery such as uncomplicated appendectomy or cholecystectomy (>6 months ago). - Severe gynaecological prolapse (grade III) - Cancer and/or adjuvant treatment within the last 6 months - Within the last three months: severe hypo- or hyperkalemia, narcosis, analgosedation, endoscopic procedure of the gastrointestinal tract, abdominal trauma - Within the last three months: gastrointestinal tract infection, food intoxication | |
| **Vision** | |
| - Inability to perform any structural retinal or functional visual assessments, for both eyes. | |
| **Imaging** | |
| - Pregnant or breastfeeding women - MR incompatible implants in the body (e.g. prosthesis, pacemakers, implanted heart valves) - Any risk of having metal particles in the eyes due to manual work without proper eye protections - Tattoos containing red pigments that form a safety risk | |
